# NRF2 plays a critical role in mitigating lipid peroxidation and ferroptosis

**DOI:** 10.1016/j.redox.2019.101107

**Published:** 2019-01-11

**Authors:** Matthew Dodson, Raul Castro-Portuguez, Donna D. Zhang

**Affiliations:** a Department of Pharmacology and Toxicology, College of Pharmacy, University of Arizona, Tucson, AZ, USA, 85721; b Arizona Cancer Center, University of Arizona, Tucson, AZ, USA, 85724

## Abstract

The transcription factor nuclear factor erythroid 2-related factor 2 (NRF2) is a key regulator of the cellular antioxidant response, controlling the expression of genes that counteract oxidative and electrophilic stresses. Many pathological conditions are linked to imbalances in redox homeostasis, illustrating the important role of antioxidant defense systems in preventing the pathogenic effects associated with the accumulation of reactive species. In particular, it is becoming increasingly apparent that the accumulation of lipid peroxides has an important role in driving the pathogenesis of multiple disease states. A key example of this is the recent discovery of a novel form of cell death termed ferroptosis. Ferroptosis is an iron-dependent, lipid peroxidation-driven cell death cascade that has become a key target in the development of anti-cancer therapies, as well as the prevention of neurodegenerative and cardiovascular diseases. In this review, we will provide a brief overview of lipid peroxidation, as well as key components involved in the ferroptotic cascade. We will also highlight the role of the NRF2 signaling pathway in mediating lipid peroxidation and ferroptosis, focusing on established NRF2 target genes that mitigate these pathways, as well as the relevance of the NRF2-lipid peroxidation-ferroptosis axis in disease.

## Highlights

• NRF2 is an important transcriptional regulator of anti-ferroptotic genes.
• NRF2 target genes prevent lipid peroxidation and the accumulation of free iron.
• Knockdown of NRF2 is sufficient to sensitize cancer cells to ferroptosis.
• Pharmacological inhibition of NRF2 can be exploited in combination with ferroptosis inducers.

## Graphical abstract

**Figure:**
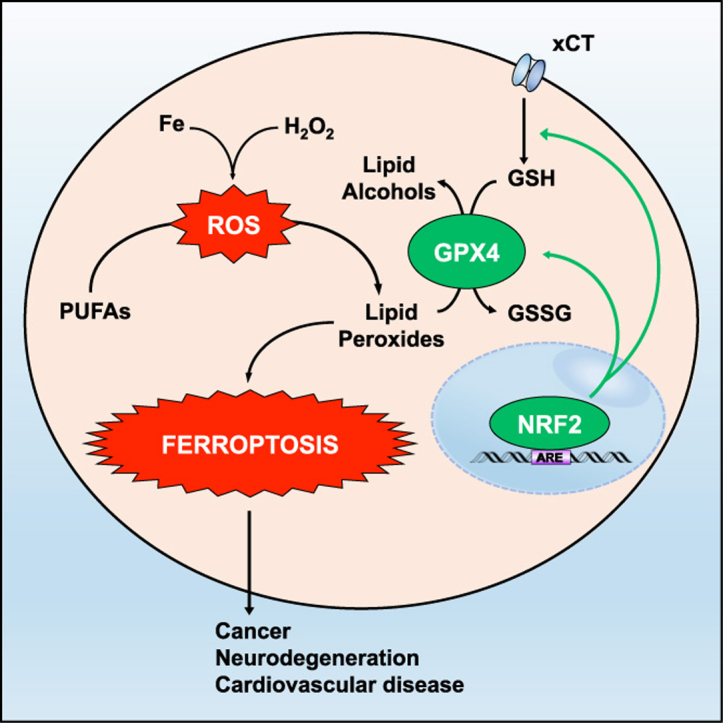

## 1 Introduction

The redox status of the cell is dictated by a balance between the generation of reactive species and their subsequent reduction by a host of antioxidant defense systems. Reactive species, such as reactive oxygen species (ROS), reactive nitrogen species (RNS), and reactive lipid species (RLS), play a critical role in mediating normal cellular physiology and signaling events; however, an excess of reactive species has been associated with the pathogenesis of many diseases. A critical aspect of excessive reactive species production is their effect on macromolecular targets, as ROS, RNS, and RLS can target nucleic acids, carbohydrates, lipids, and proteins leading to cellular dysfunction. One key example of this phenomenon is lipid peroxidation, a process by which ROS and RNS react with polyunsaturated fatty acids (PUFAs) in plasma and organellar membranes to generate lipid peroxides. Among the RLS generated by lipid peroxidation are 4-hydroxy-2-nonenal (4-HNE) and malondialdehyde (MDA), which have been linked to the progression of numerous diseases, including cancer, diabetes, neurodegeneration, cardiovascular disease, and liver disease [1]. Importantly, the cell has a number of endogenous antioxidant defense systems in place to keep the level of reactive species in check, including the transcription factor nuclear factor erythroid 2-related factor 2 (NRF2). NRF2 is considered a master regulator of the antioxidant response, as many of its downstream target genes are involved in preventing or correcting redox imbalances in the cell. While the role of NRF2 in maintaining proper redox homeostasis is well established, it has also been shown to play a critical role in mediating other key metabolic pathways, including proteostasis, xenobiotic/drug metabolism, iron/heme metabolism, carbohydrate and lipid metabolism, and apoptosis, with dysregulation of the NRF2 pathway contributing to the development or progression of a wide array of pathologies [2]. As such, proper NRF2 functionality is critical for cell survival, particularly during increased oxidative or metabolic stress.

Due to its multifaceted role in promoting cell survival, it is not surprising that inhibition of NRF2, or many of its downstream target genes, is associated with a decreased responsiveness to cellular stressors and increased cell death. A perfect example of this is the recent identification of a novel cell death cascade termed ferroptosis, a pathway of iron-dependent, lipid peroxide-induced cell death, which was initially discovered via high throughput screening of anti-tumor agents [3], [4]. Interestingly, the first two ferroptosis-inducing agents identified in these studies, RSL-3 and erastin, initiate the ferroptotic cascade via inhibition of glutathione peroxidase 4 (GPX4) and the cystine/glutamate transporter system xC^-^/xCT, respectively, both of which are downstream targets of NRF2. Even though ferroptotic cell death has been gaining a great deal of interest as a target for cancer therapies, its association with increased iron and lipid peroxide levels, as well as NRF2 dysfunction, make it a relevant cascade across a number of metabolic disease states. In this review, we will briefly summarize the process of lipid peroxidation and introduce key players in the ferroptotic cascade. Furthermore, we will highlight the role of NRF2 in mitigating both lipid peroxidation and ferroptosis, with a particular emphasis on the specific NRF2 target genes that are involved, how the function of NRF2 targets are modified by reactive lipids, as well as the relevance of this interaction in disease, including current therapeutic targets to treat diseases where these pathways are dysregulated.

## 2 Overview of lipid peroxidation and ferroptosis

As mentioned above, lipid peroxidation is a prevalent feature of a number of disease states and a key initiator of the ferroptotic cascade. Lipid peroxidation occurs in three phases: initiation, propagation, and termination (reviewed in detail in [5]). Initiation involves the abstraction of a hydrogen atom from an allylic carbon, particularly in membrane polyunsaturated fatty acids (PUFAs), by ROS, RNS, and RLS to form a lipid radical (L•). Two notable ROS initiators of lipid peroxidation are the hydroxyl radical (OH•) and hydroperoxyl radical (OOH•), which are formed via the Fenton reaction, i.e. the interaction of ferrous iron (Fe^2+^) with hydrogen peroxide (H_2_O_2_). Lipid peroxidation can also be initiated by RNS such as peroxynitrite (ONOO^-^), which is formed as a result of nitric oxide (NO•) interacting with superoxide (O_2_•^-^). During the propagation phase, the newly formed lipid radical rapidly reacts with oxygen to form a peroxyl radical (LOO•), which can then abstract another allylic carbon to form a new L• and lipid peroxide (LOOH). The propagation stage continues until termination, in which two lipid or peroxyl radicals are present at high enough concentrations to interact with one another, or endogenous antioxidants (i.e. vitamin E or GSH) donate a hydrogen atom to form a stable non-radical product. Lipid peroxides produced during the propagation phase can be reduced by glutathione peroxidases (i.e. GPX4) to lipid alcohols (LOOH), or degrade into hydroxy fatty acids or reactive aldehydes, such as MDA. These RLS can then initiate lipid peroxidation themselves, dictate cell signaling events through the modification of key proteins, or cause toxicity and initiate cell death cascades [6]. Initiation of ferroptosis occurs as a result of an accumulation of lipid peroxides and their reactive degradation products, although the mechanism by which ferroptotic death actually occurs remains unclear [7]. Key components of the ferroptotic cascade will be discussed in detail below.

**Fig. 1:**
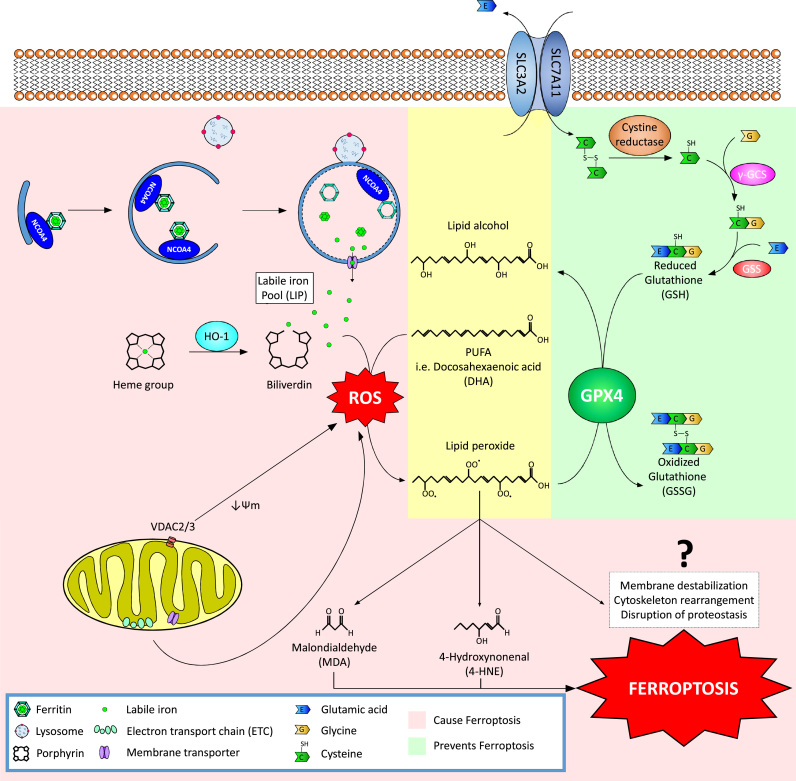
The ferroptotic cascade. Accumulation of free iron is a key initiator of ferroptosis. Free iron exists as part of the labile iron pool (LIP) and can accumulate due to altered iron import/export, decreased iron storage, or breakdown of iron-containing proteins. One recently identified pathway of free iron accumulation is through the autophagic degradation of ferritin, which stores intracellular iron, a process termed ferritinophagy. Nuclear receptor coactivator 4 (NCOA4) has been shown to act as a cargo receptor that binds to the heavy chain of ferritin delivering it to the early-stage autophagosome, resulting in degradation and release of free iron into the cytosol. Free iron can then interact with ROS (specifically hydrogen peroxide to form hydroxyl/peroxyl radicals via the Fenton reaction), which can then abstract a hydrogen atom from poly-unsaturated fatty acids (PUFAs), forming a lipid radical that rapidly reacts with oxygen to generate a lipid peroxide. Normally, lipid peroxides and their degradation products are held in check by GSH-based redox reactions. The xCT antiporter (consisting of two subunits SLC7A11 and SLC3A2) exports glutamine and imports cystine into the cell. Inside the cell, the cystine is reduced to cysteine by cystine reductase and then glutamate-cysteine ligase (GCL) and glutathione synthetase (GSS) add L-glutamate and glycine, respectively, to produce GSH. Many redox enzymes use GSH, including glutathione peroxidase 4 (GPX4), which reduces reactive aldehydes to their alcohol form. If GPX4 or xCT are genetically disrupted or pharmacologically inhibited, lipid peroxides and their degradation products accumulate, and initiate ferroptosis through a poorly understood mechanism involving membrane destabilization, cytoskeletal changes, and altered proteostasis.

Ferroptosis is an iron-dependent, lipid peroxide-driven form of cell death that is mechanistically and phenotypically distinct from other cell death processes. While the term “ferroptosis” was coined in 2012, much of the key evidence supporting this mode of cell death has been accumulating over the past few decades [8]. There are three main components that define ferroptotic cell death, an increase in free iron, accumulation of lipid peroxides, and a death phenotype that is morphologically distinct from autophagic, apoptotic, or necrotic cell death [9]. Free iron exists as part of the labile iron pool (LIP), with changes in the LIP resulting from increased uptake, decreased storage, breakdown of iron-containing proteins, or malfunction of iron exporters [10]. As discussed earlier, ferrous iron can react with hydrogen peroxide to form hydroxyl radicals, which in turn react with PUFAs to form lipid peroxides, making an abundance of free iron a critical initiator of ferroptosis (Fig. 1). The majority of intracellular iron can be found in heme-containing and mitochondrial proteins (in the form of iron-sulfur clusters) or stored as ferric iron (Fe^3+^) by ferritin. Interestingly, degradation of ferritin occurs via a process termed “ferritinophagy”, or the autophagic degradation of ferritin by nuclear receptor coactivator 4 (NCOA4) [11]. Accordingly, genetic knockdown of *NCOA4* has been shown to prevent erastin-induced ferroptosis, whereas, overexpression of NCOA4 alone was sufficient to elicit a ferroptotic phenotype [12]. Thus, tight regulation of heme, the mitochondria, and ferritin are all critical mediators of the LIP and subsequent lipid peroxide formation (Fig. 1).

**Fig. 2:**
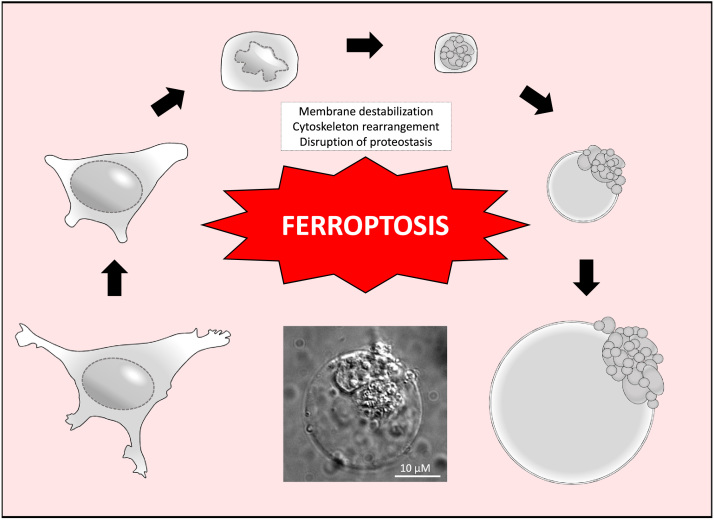
Morphological stages of Ferroptotic cell death. The exact mechanisms underlying the phenotypic changes that occur during ferroptosis remain unclear; however, it is morphologically distinct from other forms of cell death (i.e. apoptosis, necrosis, necroptosis, and autosis). Following treatment with a pro-ferroptotic agent, an initial cell shrinking is followed by condensation of cytoplasmic constituents and a “ballooning” phenotype, which involves the formation of a clear, rounded morphology consisting mainly of empty cytosol. The significant alterations to cell morphology presumably involve plasma membrane thinning/destabilization, significant cytoskeletal rearrangements, and a general disruption of proteostasis. Image represents the final “ballooning” phenotype of SKOV3 (ovarian carcinoma cell line) cells following treatment with 2.5 mM sulfasalazine for 24 h.

One of the most important components of the cell's antioxidant defenses is glutathione (GSH). GSH is a simple tripeptide consisting of glutamate, cysteine, and glycine, with the reactive thiol group on cysteine playing a critical functional role in the reduction of oxidized intracellular components. The cell obtains its cysteine via import of cystine (the oxidized dimer form of cysteine) from the extracellular environment via a cystine/glutamate antiporter dubbed system xC^-^/xCT. Imported cystine is then reduced via cystine reductase and used by two enzymes, glutamate-cysteine ligase (GCL) (previously known as gamma glutamyl-cysteine synthetase (γ-GCS)), and glutathione synthetase (GSS), to generate GSH (Fig. 1). Importantly, as mentioned previously, GSH is used by GPX4 to reduce lipid peroxides to their alcohol form, an important step in preventing ferroptosis [13] (Fig. 1). As such, it is not surprising that two of the first identified ferroptosis-inducing compounds, erastin and RSL3, are xCT and GPX4 inhibitors, respectively. GPX4 inhibition is associated with increased expression of both cyclooxygenases (i.e. COX-2) and lipoxygenases (i.e. ALOX15), which are involved in the synthesis of prostaglandins and enzymatic peroxidation of membrane PUFAs, respectively. Accordingly, both COX-2 and ALOX15 are markers of increased lipid peroxidation and ferroptotic cell death [13], [14]. Aside from direct inhibition of GPX4, GSH depletion (i.e. via buthionine sulfoximine) has also been shown to initiate ferroptosis [15]. Similarly, low levels of the reducing agent NADPH and the enzyme acyl-CoA synthetase long chain family member 4 (ACSL4), which metabolizes lipid peroxides, are both biomarkers correlated with an enhanced sensitivity to ferroptosis [16], [17]. Aside from increases in free iron, and increased lipid peroxidation, the final critical feature of ferroptosis is morphological, and involves a destabilization of the plasma membrane, disrupted proteostasis, and cytoskeletal rearrangements that result in a distinct “ballooning” phenotype [18], [19], [20], [21] (Fig. 2). Less is known about the exact mechanisms that drive the ballooning phenotype, with further studies being needed. Importantly, many of the key anti-ferroptotic pathway components are under the transcriptional control of NRF2. Therefore, NRF2 has emerged as a key regulator of both lipid peroxidation and ferroptosis, which will be discussed in the following section.

## 3 The role of NRF2 in preventing lipid peroxidation and ferroptosis

**Fig. 3:**
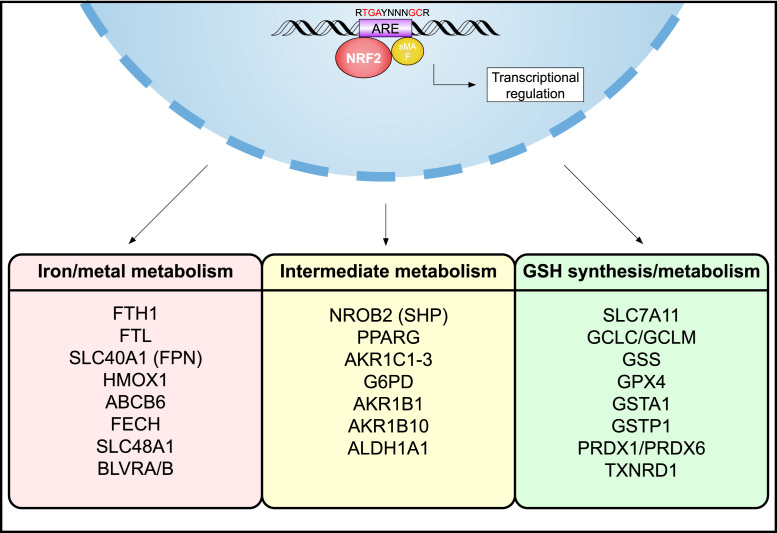
NRF2 target genes are involved in preventing lipid peroxidation and ferroptosis. NRF2 is a master regulator of the antioxidant response and has been shown to regulate the activity of several ferroptosis and lipid peroxidation-related proteins. These targets can be divided into three broad classes: iron/metal metabolism, intermediate metabolism, and GSH synthesis/metabolism. Examples of each class are included, all of which have a verified antioxidant response element (ARE).

NRF2 is a stress-inducible transcription factor, whose protein levels are kept basally low by three different E3-ubiquitin ligase complexes: Kelch-like ECH-associated protein 1-Cullin 3-Ring box 1 (KEAP1-CUL3-RBX1), S-phase kinase-associated protein 1-Cullin1-Rbx1/β-transducin repeat-containing protein (SCF/β-TrCP), and synoviolin/Hrd1. Should degradation of NRF2 by any of these complexes be compromised, as a result of genetic mutations, endogenous stress-induced modifications, competitive binding of other interacting partners, or exogenous pharmacological inhibition, NRF2 can then translocate to the nucleus to initiate the transcription of antioxidant response element (ARE)-containing genes. Notably, many of the proteins and enzymes responsible for preventing lipid peroxidation, and thus initiation of ferroptosis, are NRF2 target genes. These can be broken down into three broad categories based on their function: iron/metal metabolism, intermediate metabolism, and glutathione synthesis/metabolism (Fig. 3).

NRF2 targets play a critical role in mediating iron/heme metabolism [22]. Both the light chain and heavy chain of ferritin (*FTL*/*FTH1*), the key iron storage protein, as well as ferroportin (*SLC40A1*), which is responsible for iron efflux out of the cell, are controlled by NRF2 [23], [24]. Furthermore, heme-oxygenase 1 (*HMOX1*), the enzyme that catalyzes the conversion of heme into biliverdin; ferrochelatase (*FECH*) and ATP-binding cassette sub-family B member 6 (*ABCB6*), critical components of heme synthesis; and solute carrier family member 48 member A1 (*SLC48A1*), a heme transporter, are all upregulated by NRF2 [25], [26], [27], [28]. Biliverdin reductase A and B (*BLVRA*/*B*) have also been shown to be directly upregulated by NRF2 activation [23], indicating the essential link between NRF2 function and iron/heme homeostasis.

Aside from iron and heme, NRF2 regulates a host of metabolites involved in intermediate metabolism, many of which are important for the catabolism/detoxification of reactive intermediates and for regenerating NADPH, a key electron donor needed for reduction of oxidized substrates. For example, NRF2 targets are involved in lipid metabolism (i.e. small heterodimer partner (SHP), *NROB2*; and peroxisome proliferator-activated receptor γ, *PPARG*), the reduction of aldehydes and ketones to their alcohol forms (i.e. aldo-ketoreductases, *AKR1C1–3*; *AKR1B1* and *AKR1B10*), oxidation of aldehydes to their carboxylic acid form (aldehyde dehydrogenase 1 family member A1, *ALDH1A1*), and glucose metabolism/NAPDH regeneration (i.e. glucose-6-phosphate dehydrogenase, *G6PD*) [29], [30], [31], [32]. Finally, a number of integral glutathione synthesis and metabolism related enzymes are under the control of NRF2 including, both the catalytic and modulatory subunits of glutamate-cysteine ligase (*GCLC/GCLM*), glutathione synthetase (*GSS*), and a subunit of the cystine/glutamate transporter xCT (*SLC7A11*), all of which are indispensable for GSH synthesis [33], [34], [35], [36], [37]. Also, a number of redox enzymes that utilize GSH or NADPH to reduce oxidized substrates (i.e. glutathione-S-transferases pi 1 and alpha 1, *GSTP1* and *GSTA1*; peroxiredoxins 1 and 6, *PRDX1* and *PRDX6*; and thioredoxin reductase, *TXNRD1*) are also targets of NRF2 [38]. As discussed in detail in the prior section, glutathione peroxidase 4 (*GPX4*), which is also an established NRF2 transcriptional target [39], [40], is an integral anti-ferroptotic reducer of lipid peroxides. Thus, NRF2 is a critical mitigator of both lipid peroxidation and ferroptosis, inferring that diseases associated with increased lipid peroxides and ferroptosis could be a result of aberrant NRF2 signaling. In the following sections, the modification of NRF2 signaling proteins by reactive lipids, as well as the role of NRF2, lipid peroxidation, and ferroptosis in driving metabolic diseases will be explored.

## 4 Lipoxidation of NRF2 signaling pathway components

Importantly, while many NRF2 target genes are involved in preventing the formation of lipid peroxides and the progression of the ferroptotic cascade, a number of proteins involved in the NRF2 signaling pathway are also direct targets of lipoxidation themselves. Perhaps the most notable is KEAP1, the negative regulator of NRF2, which contains a number of cysteine residues that can undergo electrophilic attack, including C151, C273, and C288 [41]. A number of reactive lipid species, including 4-HNE and the cyclopentenone prostaglandin 15-deoxy-Δ(12,14)-prostaglandin J2 (15d-PGJ2), have been shown to adduct to KEAP1 cysteines and activate NRF2 target gene expression [42]. While electrophilic modification of KEAP1 is a well-established activator of the NRF2 response, a number of proteins encoded by NRF2 target genes are also susceptible to lipoxidation. For example, peroxisome proliferator-activated receptor-γ (*PPARG*) is activated by 15d-PGJ2 binding to C285 in the ligand-binding domain, which results in a conformational change that facilitates the recruitment of cofactors and activation of PPAR-γ driven transcription [43]. Conversely, GCLC, GCLM, PRDX6, TXNRD1, GSTP1, and GSTA1, all of which are NRF2 targets responsible for maintaining redox homeostasis, as well as BLVRA, which is involved in heme catabolism, can all be modified by either 15d-PGJ2 or 4-HNE, inhibiting their enzymatic activity [44], [45], [46], [47], [48], [49], [50], [51]. This infers that while electrophilic modification of KEAP1 can activate NRF2 to prevent lipid peroxidation and ferroptosis, a number of reactive lipid species can suppress the function of NRF2 target genes, which could in turn play an important role in initiating the ferroptotic cascade.

Other NRF2 downstream targets that can be modified by reactive lipid species include AKR1B1 and AKR1B10, which as mentioned above are responsible for reducing reactive aldehydes to their less toxic alcohol forms. 4-HNE and prostaglandin A1 can directly modify AKR1B1 and AKR1B10, respectively [52], [53]. Furthermore, ALDH1A1 exhibits reduced activity in the presence of acrolein, a lipid peroxidation product [54]. These results indicate that excess production of reactive lipids can significantly hinder the enzymes that detoxify them. Thus, it is important to note that not only is NRF2 critical in preventing the formation of reactive lipid intermediates, but its downstream effectors can also be modified by electrophilic lipid species, inferring an intimate relationship between pathway activation and preventing the pathological effects associated with increased lipid peroxidation, including ferroptosis. As such, it is worthwhile to note that in a disease setting where NRF2 is high, such as cancer, tumor cells could utilize these protective detoxification systems to prevent lipid peroxide accumulation and lipoxidation of target proteins to survive; whereas in diseases where NRF2 is low, such as neurodegenerative diseases, increased lipoxidation and inactivation of downstream NRF2 targets could significantly enhance overall protein lipoxidation and ferroptosis, furthering disease progression. Hence, targeting NRF2 to modulate lipid peroxidation and ferroptosis is a viable strategy for disease intervention, which will be discussed below.

## 5 Targeting NRF2 to induce lipid peroxidation and ferroptosis as a cancer therapy

Mentioned above, ferroptosis was initially discovered in studies utilizing high throughput screening to identify anti-tumor agents capable of killing oncogenic RAS transformed cell lines [3], [4]. These studies indicate that pro-ferroptotic agents could be a particularly powerful tool for treating transformed cancer cells that are resistant to pro-oxidative and pro-apoptotic therapeutics. Another example of this is p53-driven cancers, where p53 is either mutated or functionally inactive. It has been shown that wild type p53 inhibits cystine uptake via system xCT, and this inhibition requires its downstream target p21 (*CDKN1A*) [55]. This infers that tumors associated with a loss of p53 or mutations that decrease its interaction with xCT, could have abnormally high xCT activity, and thus an excess of glutathione, possibly making them resistant to established ferroptosis inducers. Thus, finding other druggable targets in the pathway is important for treating both apoptosis and ferroptosis-resistant cancer types.

The majority of ferroptosis-inducing agents identified to this point, including sorafenib, sulfasalazine, and FIN56 are system xCT or GPX4 inhibitors, similar to the two initially identified compounds, erastin and RSL-3, respectively [56]. As expected, based on its transcriptional regulation of these two targets, as well as many other key pathways, inhibition of NRF2 should significantly enhance the therapeutic efficacy of pro-ferroptotic agents. Indeed, treatment with trigonelline has been shown to enhance sorafenib-induced hepatocellular carcinoma cell death [57] and artesunate-induced ferroptosis in head and neck cancer cell lines [58]. Accordingly, the level of NRF2 has been directly correlated with ferroptosis sensitivity, as increased expression of NRF2 prevents ferroptosis, whereas decreased NRF2 enhances the sensitivity of cancer cells to pro-ferroptotic agents [57], [59]. These studies indicate inhibitors of NRF2, as well as its downstream targets, could be viable targets to induce ferroptosis-dependent cancer cell death. However, it is important to note that trigonelline's pro-ferroptotic effects may not be specific to NRF2 inhibition, as it significantly alters proteostasis, and as such could induce ferroptosis through pathways independent of NRF2 and the GSH-system. Despite this fact, targeting NRF2 and its downstream targets remains a viable approach for utilizing ferroptosis to kill resistant cancer cells until a more specific inhibitor of NRF2 or other druggable downstream ferroptotic pathway targets are identified.

It is also important to note that a number of disease states, including neurodegeneration and cardiovascular disease have been associated with increased ferroptosis [19], [60], [61]. In the context of these diseases, the goal is to prevent ferroptotic death through the use of iron chelators (i.e. deferoxamine and cyclipirox), inhibitors of lipid peroxidation (i.e. ferrostatin and liproxstatin), or activators of NRF2 (i.e. sulforaphane and dimethyl fumarate). For example, ferrostatin has been shown to protect against ischemic injury in the brain, liver, kidney, and heart and reduce neurodegenerative phenotypes in a number of cell and animal models, as well as prevent excess iron-induced ferroptosis in cardiomyocytes [56], [62], [63]. Therefore, both the activation and inhibition of ferroptosis, particularly utilizing pharmacological modulation of the NRF2 signaling pathway, could both prove effective depending on the disease context.

## 6 Conclusions and future directions

As a deeper mechanistic understanding of ferroptosis and its relevant components continues to grow, the pivotal role NRF2 plays in mediating this process becomes more apparent. Of particular importance is the fact that the antioxidant, iron, and intermediate metabolic status of the cell can all be mediated by NRF2 target genes. Two of the most critical targets whose inhibition initiates ferroptosis, xCT and GPX4, are well established to be regulated by NRF2. As such, targeting NRF2 in diseases where lipid peroxidation and ferroptosis are prevalent features remains an extremely viable approach. However, very little is known regarding the downstream effectors of ferroptosis that mediate the lipid peroxide-induced cell death cascade, which themselves could be regulated in some manner by NRF2. Thus, targeting the upstream regulators of the ferroptotic cascade, including dysregulated iron levels and the formation of ROS, RNS, and RLS, through pharmacological modulation of the NRF2 signaling pathway remains one of the more optimal approaches for treating ferroptosis-related pathologies.
